# Unexpected observation of splitting of skyrmion phase in Zn doped Cu_2_OSeO_3_

**DOI:** 10.1038/srep13579

**Published:** 2015-09-09

**Authors:** H. C. Wu, T. Y. Wei, K. D. Chandrasekhar, T. Y. Chen, H. Berger, H. D. Yang

**Affiliations:** 1 Department of Physics, National Sun Yat-Sen University, Kaohsiung, 804 Taiwan; 2 Institute of Physics of Complex Matter, Ecole Polytechnique Federal de Lausanne, CH-1015 Lausanne, Switzerland

## Abstract

Polycrystalline (Cu_1−x_Zn_x_)_2_OSeO_3_ (0 ≤ x ≤ 0.2) samples were synthesized using solid-state reaction and characterized by X-ray diffraction (XRD). The effect of Zn doping upon saturation magnetization (M_S_) indicates that the Zn favors to occupying Cu(II) square pyramidal crystallographic site. The AC susceptibility (χ′_ac_) was measured at various temperatures (χ′_ac_–*T*) and magnetic field strengths (χ′_ac_–H). The Zn doping concentration is found to affect greatly the M-*T* and χ′_ac_-*T*. The skyrmion phase has been inferred from the χ′_ac_-H data, and then indicated within the H-*T* phase diagrams for various Zn doping concentrations. The striking and unexpected observation is that the skyrmion phase region becomes split upon Zn doping concentration. Interestingly, second conical boundary accompanied by second skyrmion phase was also observed from dχ′_ac_/dH vs. H curves. Atomic site disorder created by the chemical doping modulates the delicate magnetic interactions via change in the Dzyaloshinskii-Moriya (DM) vector of distorted Cu(II) square pyramidal, thereby splitting of skyrmion phase might occur. These findings illustrate the potential of using chemical and atomic modification for tuning the temperature and field dependence of skyrmion phase of Cu_2_OSeO_3_.

Ever since the discovery of high-*T*_c_ superconductivity^1^, copper oxide materials have garnered significant attention due to exotic physical properties such as charge order stripes^2^, electronic phase separation^3^, giant magnetoresistance^4^ and multiferroics^5^. This family of materials produce complex and rich phase diagrams because of the strong interplay between their crystal lattices and the spin and orbital degrees of freedoms^4^. The prime research focus is to address the underlying mechanism behind the physical insight and to establish the technological relevance of these materials.

Recently, a peculiar magnetic state called as “skyrmion or A-phase” has been stabilized in noncentrosymmetric B20 chiral magnetic systems, such as MnSi, FeSi and FeGe^6^,^7^,^8^. Magnetic skyrmion is a topologically stable particle-like spin configuration where spins mold into a vortex-like ordering. More recently, the recognition of skyrmion in insulating spin-1/2 Cu_2_OSeO_3_^9^ was reported. It has further triggered the intensive research activity because skyrmion motion can be controlled by external electric fields instead of Joule heating currents, thus making it a good candidate for ultra-low power spintronic devices^9^,^10^. The skyrmion lattice can be probed with several experimental methods, such as Lorentz transmission electron microscopy^11^, magnetic force microscopy^12^, spin-polarized scanning tunneling microscopy^13^ and small angle neutron scattering^14^. The signature of skyrmion lattice can also be detected by relatively simple methods such as magnetic χ′_ac_^15^, heat capacity^16^, topological Hall effect^17^ and electrical polarization^18^,^19^,^20^.

From the crystallographic view, the common point to both high-*T*_c_ superconductor YBa_2_Cu_3_O_7_ and skyrmion Cu_2_OSeO_3_ systems is the presence of two different Cu sites. It indicates the microscopic picture of these novel phenomena are hidden in the local structures of two Cu ions and its complex magnetic interactions between Cu(I)/Cu(II) ions. In YBa_2_Cu_3_O_7_, Cu ions are located on two different sites i.e. Cu(I)O chains and Cu(II)O_2_ planes. The superconductivity mainly originates from the electron transport across the Cu(II)O_2_ planes^21^. In Cu_2_OSeO_3_, the two Cu^2+^ ions occupy trigonal bipyramidal (Cu(I)) and square pyramidal (Cu(II)) of oxygen ligands, with the ratio of 1:3^22^. Neutron scattering, μSR, and NMR experiments have established the ferrimagnetism of Cu spin with three up (Cu(II)) and one down (Cu(I)) spin alignment^22^,^23^,^24^. However, a close inspection of the magnetic interactions and the crystal lattice of Cu^2+^ spins reveal the presence of more complex superexchange interactions between the Cu-O-Cu bridges^25^,^26^. Interestingly, unexpected two distinct coupled skyrmion sublattices, which arise from the two different magnetic active orbitals i.e. Cu(I) and Cu(II), have been identified in Cu_2_OSeO_3_ system using the orbital sensitive resonant X-ray scattering^27^. It indicates that the site-specific chemical (Cu(I)/Cu(II)) doping might help to gain further insights of this novel skyrmion phase. Following the similar study in high-*T*_c_ YBa_2_Cu_3_O_7_^21^, we explore the chemical doping to shed the light on the microscopic origin of these two skyrmion sublattices. The primary goals of this work are to find out what is the role of Cu(I) and Cu(II) sites in the formation of skyrmion phase? What are the effects of nonmagnetic Zn doping on the skyrmion lattice ? Consequently, what are the electronic and magnetic nature of skyrmion phase in Cu_2_OSeO_3_?

## Results and Discussion

**Figure 1:**
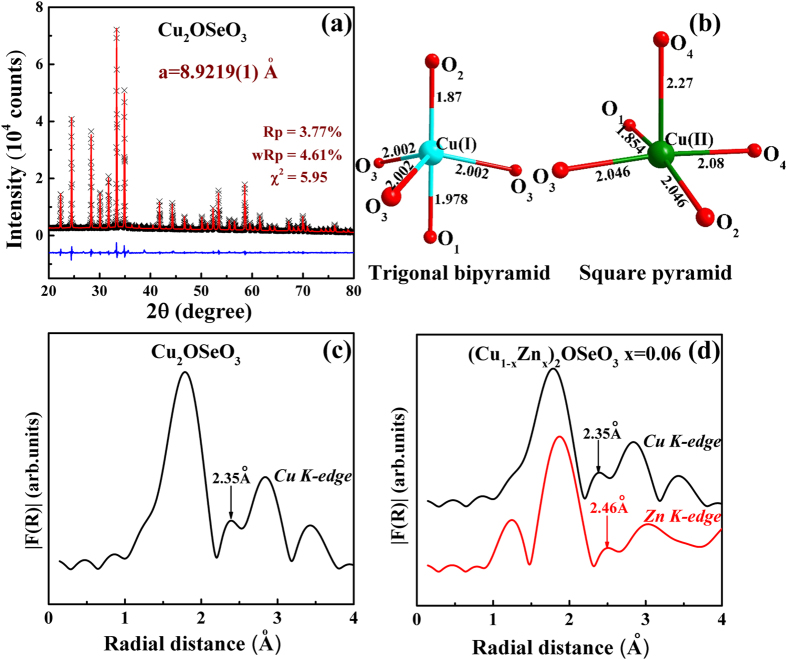
(**a**) Rietveld refinement of X-ray pattern of Cu_2_OSeO_3_ sample. (**b**) Trigonal bipyramidal and square pyramidal sites of Cu_2_OSeO_3_ derived from Rietveld analysis. Fourier transforms moduli radial distribution functions of EXAFS spectra (**c**) Cu *K*-edge for Cu_2_OSeO_3_. (**d**) Cu and Zn *K*-edge for (Cu_0.94_Zn_0.06_)_2_OSeO_3_ respectively. Arrows near small hump in Fig. 1 (c,d) indicate Cu(II)-O_4_ and Zn-O_4_ bond lengths respectively.

Figure 1(a) shows the X-ray diffraction pattern of Cu_2_OSeO_3_ sample. The pattern showed the cubic crystal structure and refined with P2_1_3 space group using GSAS Rietveld program. The obtained lattice parameter (a = 8.9219(1) Å) consistent with the previous report indicates the good quality of the sample^22^. The local structures trigonal bipyramidal and square pyramidal (represented in Fig. 1(b)) determined by the Rietveld method are highly distorted similarly to previous report^22^. To check the Zn occupation in Cu_2_OSeO_3_ crystal, we have performed the room temperature extended X-ray absorption fine structure (EXAFS). EXAFS is highly sensitive technique to probe local crystal structure such as coordination number of ligand and interatomic distance of metal ion to ligand atoms^28^. The EXAFS spectrum analysis was carried out in Athena software. The moduli of their Fourier transforms |F(R)| are presented in Fig. 1(c,d). As seen from Fig. 1(c), the spectrum for Cu_2_OSeO_3_ at Cu *K*-edge shows the prominent peak around 1.85 Å followed by the small hump at 2.35 Å. To understand the EXAFS spectrum we have compared the EXAFS spectrum with Fig. 1(b). The primary difference between these two structures of Fig. 1(b) is the axial bond length that is longer for the square pyramidal structure. By comparing EXAFS spectrum with the bond lengths of Fig. 1(b), the small hump can be assigned to the axial bond length of Cu(II)-O_4_ square pyramidal. Fig. 1(d) depicts the |F(R)| spectra for (Cu_0.94_Zn_0.06_)_2_OSeO_3_ at Cu and Zn *K*-edges. As shown in the figure the Zn *K*-edge spectrum qualitatively identical to the Cu *K*-edge (low radial distance peak might come from other sources such as atomic XAFS and/or multielectron excitations^28^). As expected Zn spectrum shifted to high radial distances, this indicates the enhancement of bond length due to the changes in the covalent bond formation upon Zn doping. Most importantly, Zn spectrum exhibits the Zn-O_4_ bond peak even for small doping concentration (6% of Zn) which qualitatively indicates the Zn certainly occupy the Cu(II) site. On the other hand, the low Zn doping concentration hampers the quantitative estimation of Zn at Cu(II) site.

**Figure 2:**
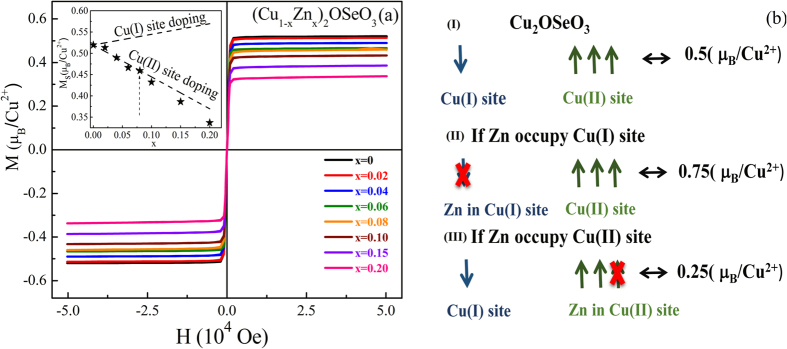
(**a**) M vs. H curves of (Cu_1−x_Zn_x_)_2_OSeO_3_ (0 ≤ x ≤ 0.2) series at *T* = 5 K; Inset shows the M_S_ vs. Zn doping concentration. Dashed lines indicate the theoretically predicted occupation probabilities of Zn at (Cu(I) or Cu(II) crystallographic positions respectively. The experimental data matches the Cu(II) site occupation. (**b**) The graphical representation of resultant magnetic moment for (I) Cu_2_OSeO_3_ (II) Zn at Cu(I) site and (III) Zn at Cu(II) site respectively.

Figure 2 depicts the isothermal M-H loops at *T* = 5 K with the field up to 5 T for (Cu_1−x_Zn_x_)_2_OSeO_3_ (0 ≤ x ≤ 0.2). The saturation magnetization M_S_ at 5 T is observed to be 0.519 μ_B_/Cu^2+^ for x = 0, a close match with the theoretical predicted (0.5 μ_B_/Cu^2+^) ferrimagnetic alignment of Cu spins^9^. For the doping case, the nonmagnetic Zn^2+^ ion might occupy two possible crystallographic (Cu(I)/Cu(II)) sites. If non-magnetic Zn replaces any one of three ferromagnetically aligned Cu(II) sites that decrease the overall magnetization of the unit cell (0.25 μ_B_/Cu^2+^). On the other hand, Zn placed at Cu(I) site enhances the overall magnetization as well as ferromagnetic strength (0.75 μ_B_/Cu^2+^). To estimate the probable Zn site occupation we have simulated the M_S_ values for both the case of Cu(I)/Cu(II)) sites. The inset also plots the theoretically predicted values that would be expected for each of doping sites. The measured values line up well with theoretical expectation for the Cu(II) sites, which reveals that the nonmagnetic Zn^2+^ ions favor occupying the Cu(II) site.

**Figure 3:**
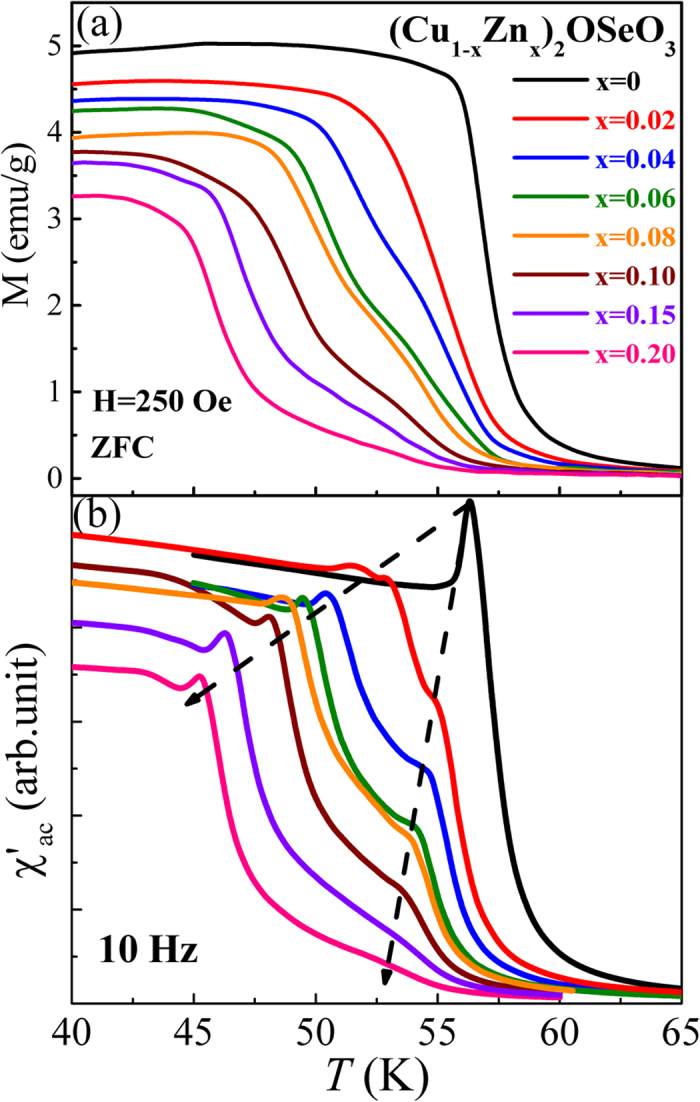
(**a**) M vs. *T* (**b**) χ′_ac_ vs. *T* curves for (Cu_1−x_Zn_x_)_2_OSeO_3_ (0 ≤ x ≤ 0.2). The dashed lines in (**b**) added as a guide to the eye for variation of magnetic transition temperatures with Zn doping.

Figure 3 shows the M-*T* and χ′_ac_–*T* curves for (Cu_1−x_Zn_x_)_2_OSeO_3_ (0 ≤ x ≤ 0.2). For x = 0, the M-*T* and χ′_ac_–*T* curves are in good agreement with those of measured in single crystal Cu_2_OSeO_3_^29^. Moreover, both M-*T* and χ′_ac_–*T* are changed correspondingly and systematically with Zn doping concentration x. Just below ferrimagnetic transition *T*_*C*_ ~ 58 K, a clear peak appeared at 56 K shown in χ′_ac_–*T* for x = 0 is the hallmark signature of skyrmion phase^30^. It is noted that the peak is lowered in temperature and becomes fainter with increasing x. On the other hand, a second smaller but notable peak is developed as x ≥ 0.02 and shifted towards lower temperature with increasing x. These results clearly hint the possible formation of second skyrmion phase when x ≥ 0.02.

**Figure 4:**
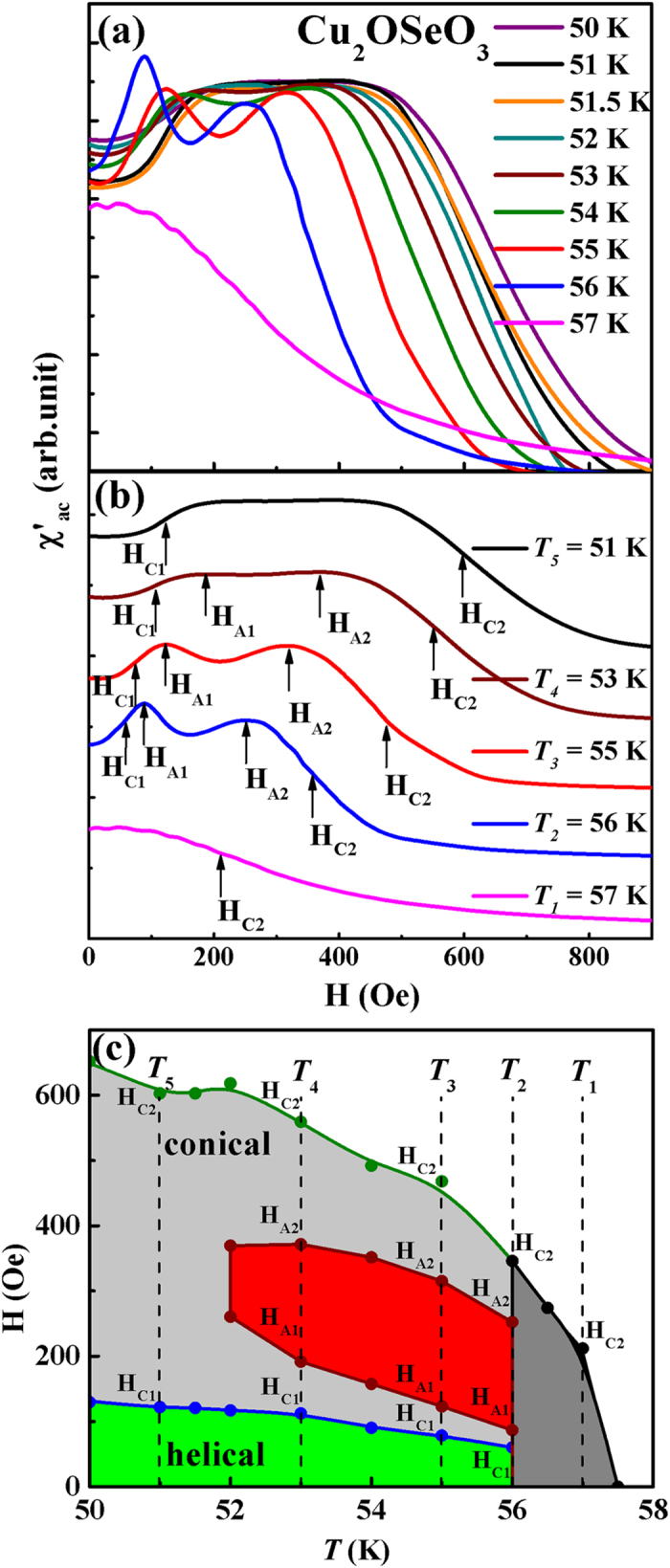
(**a**) χ′_ac_ vs. H plot at temperatures 50–57 K. (**b**) Selected temperature curves from (**a**), but offset each other for easy comparison. The notations of H_A1_ and H_A2_ indicate skyrmion phase boundaries while H_C1_ and H_C2_ indicate conical phase boundaries respectively. The values of H_A1_ and H_A2_ are determined by the peaks while H_C1_ and H_C2_ are the inflection points in the first derivative of χ′_ac_ vs. H curves. (**c**) H vs. *T* phase diagram for Cu_2_OSeO_3_, where the skyrmion phase zone is marked in red.

AC susceptibility is known to be a sensitive technique for revealing coexisting phases in complex magnetic materials, including skyrmion system^15^,^25^. The H dependent χ′_ac_ curves at 50–58 K are shown in Fig. 4(a) for Cu_2_OSeO_3_. Below the vicinity of peak temperature ~56 K as shown Fig. 3b, the evolution of peak anomalies is noticed in the intermediate field region 100 ≤ H ≤ 400 Oe in χ′_ac_ vs. H curves (Fig. 4(a)). It confirms the growth of skyrmion phase^15^,^25^ as *T* ≤ 56 K. For low temperatures, the peak anomalies suppress in the χ′_ac_ vs. H curves which indicates the skyrmion phase is almost disappeared for *T* < 52 K. To demonstrate the typical way of extracting the skyrmion phase boundaries, the χ′_ac_ vs. H curves at selected temperatures *T*_*1*_ = 57 K, *T*_*2*_ = 56 K, *T*_*3*_ = 55 K, *T*_*4*_ = 53 K and *T*_*5*_ = 51 K are shown in Fig. 4(b). The H-*T* phase diagram with skyrmion zone marked in red for Cu_2_OSeO_3_ is successfully constructed and displayed in Fig. 4(c). The evolution of different magnetic phase zones, i.e. helical, conical and skyrmion boundaries are in good agreement with previously published results^14^.

**Figure 5:**
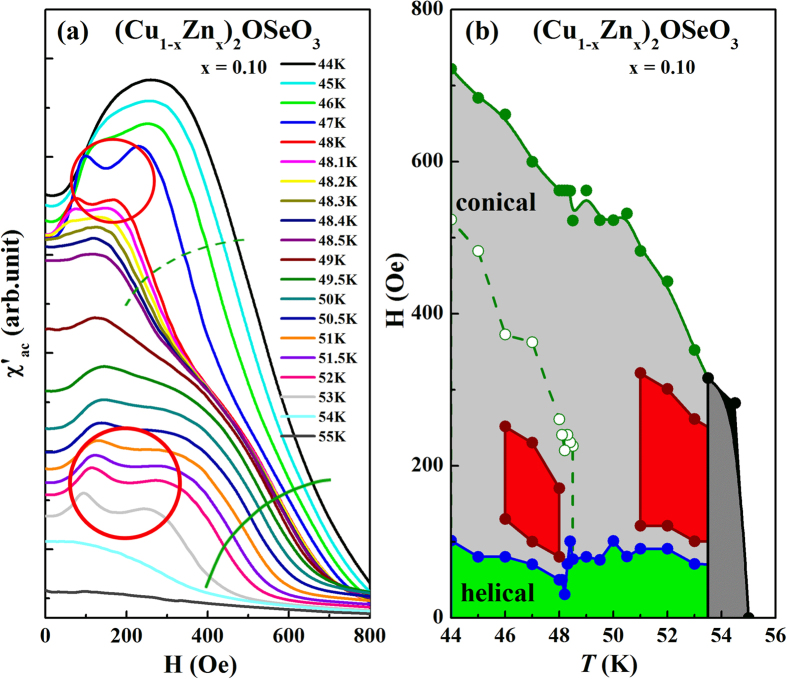
(**a**) χ′_ac_ vs. H of at temperatures 44–55 K and (**b**) corresponding H vs. *T* phase diagram for (Cu_1−x_Zn_x_)_2_OSeO_3_ (x = 0.1). The two red circles in (**a**) corresponding to respective skyrmion zones in (**b**). Solid and dashed green lines denote the conical phase boundaries.

To investigate the influence of Zn doping on the skyrmion phase of Cu_2_OSeO_3_, the χ′_ac_ vs. H for (Cu_1−x_Zn_x_)_2_OSeO_3_ (0 ≤ x ≤ 0.2) are performed for a broad range of *T*. The representative results of x = 0.1 are shown in the Fig. 5(a). The characteristic features of the χ′_ac_ vs. H curves for x = 0.1 at 51 K ≤ *T* ≤ 53 K are comparable to that of Cu_2_OSeO_3_ at 52 K ≤ *T* ≤ 56 K (shown in Fig. 4(a)), that the signature of skyrmion phase is noticed with two peaks. With decreasing temperature to 48 K < *T* < 51 K, the two peaks become smeared. However, as the temperature is lowered to 47 K ≤ *T* ≤ 48 K, the signature of skyrmion peaks reappeared for H between 80 and 210 Oe. This unexpected observation of second skyrmion phase is a quite novel phenomenon and never been reported in the Cu_2_OSeO_3_ system. Along with second skyrmion signature, χ′_ac_ displays second inflection point in the dχ′_ac_/dH vs. H (see supplementary material) curves. It might indicate the appearance of the second conical boundary in the phase diagram accompanied with the second skyrmion phase. However, further experimental verification needed to confirm these signatures. It is important to emphasize that, similar atomic doping effect in a metallic skyrmion systems such as Mn_1−x_Fe_x_Si and Mn_1−x_Co_x_Si^31^ lead to Quantum phase transitions with a suppressed of helical magnetic and skyrmion phases. Contradictory, the present study indicates the atomic disorder strongly influence the ground state magnetic properties of the Cu_2_OSeO_3_ system that lead to more complex magnetic behavior with the generation of additional novel phases in the H-*T* phase diagram. The multiple inflection points in dχ′_ac_/dH vs. H curves are systematically changes with the Zn doping concentration in a selected temperature window, which are displayed in the supplementary material. Following the same plotting procedure as mentioned in Fig. 4(b,c), the Fig. 5(b) is successfully constructed from Fig. 5(a).

**Figure 6:**
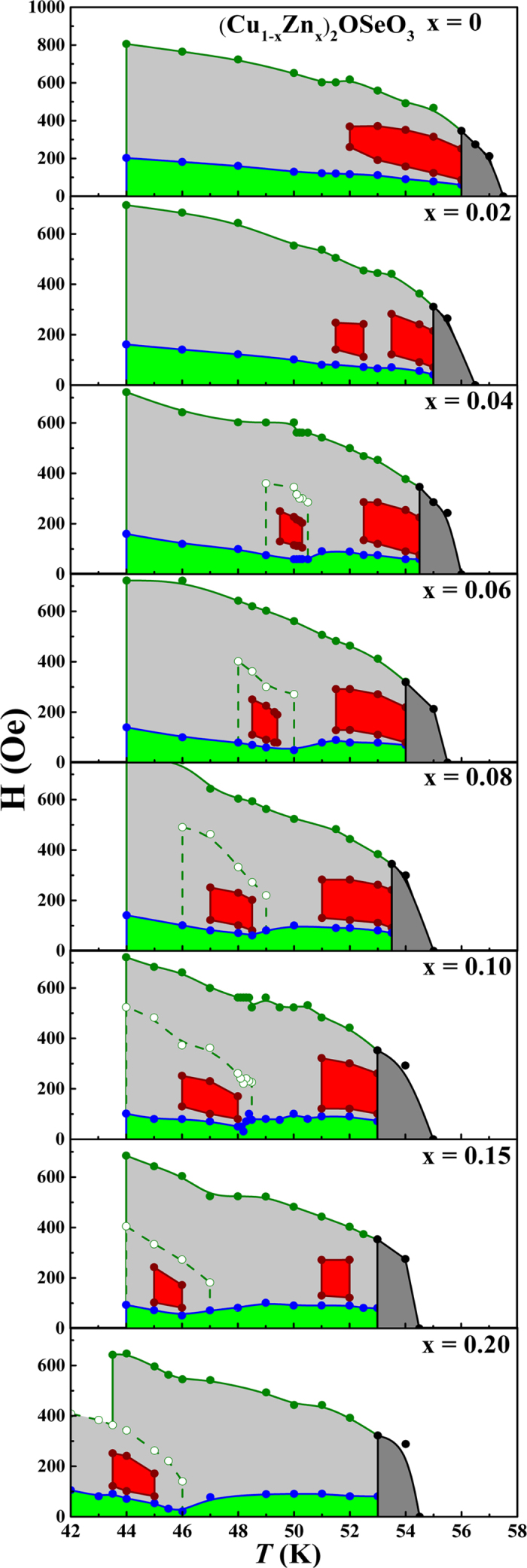
H-*T* phase diagrams of all (Cu_1−x_Zn_x_)_2_OSeO_3_ (0 ≤ x ≤ 0.2) samples. Skyrmion zone is indicated by two red areas respectively. Solid and dashed green lines denote the two conical phase boundaries respectively.

Applying the same method as described in Figs 4 and 5, the H-*T* phase diagrams derived from χ′_ac_-H data at selected temperatures for each of 8 samples (Cu_1−x_Zn_x_)_2_OSeO_3_ (0 ≤ x ≤ 0.2) are established and shown in Fig. 6, where the boundaries of conical, helical and skyrmion phases are plotted (with various colors) approximately using limited and discrete data points. The surprising finding is that the single skyrmion phase at x = 0 is splitted into two well-defined small branches in two different temperature regions and about the same magnetic field as x ≥ 0.02. Moreover, the second skyrmion phase is hosted by the second conical phase boundary. Both branches of skyrmion phase are systematically shifted towards low temperature side with x. The opening of temperature gap between two branches of skyrmion phase is larger for higher Zn doping concentration. The second skyrmion phase and its associated conical phase boundaries are firmly decoupled with that of the initial skyrmion phase; this can be clearly visible for the doping concentration x ≥ 0.1. Meanwhile, the high-temperature branch becomes harder to extract from the data as x > 0.15. In fact, the trends of splitting, suppression, and decreasing in temperature of skyrmion phases found in Fig. 6 are consistent with those observed in Fig. 3(b).

The splitting of skyrmion phase for Zn doping Cu_2_OSeO_3_ is a novel and interesting phenomenon. Similar to high-*T*_c_ superconducting materials, it might be associated with the two crystallographic sites of Cu ions and their complex magnetic interactions. From the magnetic point of view, Cu_2_OSeO_3_ exhibits a quite complex behavior with several magnetic interactions between Cu(I)/Cu(II) via oxygen bridging^25^. In the unit cell of Cu_2_OSeO_3_ structure, the 4 Cu(I) ions are placed in the undistorted trigonal bipyramidal, whereas 12 Cu(II) ions are distributed among the distorted square pyramidal sites. According to Goodenough-Kramer (G-K) rules, a negative superexchange interaction (<*J*) corresponds to the orbital overlap angle of Cu ions close to 90°, and it goes to positive (>*J*) if it deviates from 90° 32. A close examination of Cu_2_OSeO_3_ using an AC susceptibility technique exposed the antiferromagnetic (AFM) ordering at 59 K followed by ferromagnetic (FM) ordering at 58 K^25^. The complicated behaviour originates from the unequal strength of three nearest neighbors (NN) AFM interaction of Cu(I)-Cu(II) and three NN FM interaction of Cu(II)-Cu(II) ions^25^. The crystallography studies in Fig. 1 along with magnetization studies in Fig. 2 suggest that the Cu(II) is the preferable site for Zn doping. Replacing of nonmagnetic Zn^2+^ for Cu^2+^ site enhances the Coulombic repulsion of electronic orbital that leads to the decrease in *T*_*C*_. Moreover, the presence of nonmagnetic dopant along with the weak perturbation for the overlaps of electronic orbital can show a significant impact on the complex magnetic exchange interactions between Cu(I)/Cu(II) ions. In general, the helical ground state originates from the competition between Heisenberg superexchange and DM interactions of Cu ions^9^. The strength of DM interaction depends on the relative change of g-factor from the free electron g-value, i.e. DM ∝ (Δg/g)J, where J is the exchange interaction term^33^. Symmetry calculation analysis using Raman modes by Gnezdilov *et al.* indicates the change of DM strength is more significant for the distorted Cu(II) square pyramidal^33^. The disorder effect is further amplified by doping the nonmagnetic Zn that strongly modulates the DM interaction strength via the change of radial vector and the canting angle between the adjacent spin pairs. Consequently, it manipulates the complex magnetic interactions between Cu(I)/Cu(II) ions.

Similar to resonant X-ray scattering studies on Cu_2_OSeO_3_, these two skyrmion phases can be possibly associated with the two Cu sublattices^27^. Chemical doping might alters the modulation vector of Moirelike skyrmion phase of pure Cu_2_OSeO_3_. These results reminiscent the recent observation of unexpected coupled skyrmion sublattices^27^ and theoretical prediction of novel half skyrmion state in Cu_2_OSeO_3_ system^34^. However, it needs more experimental verifications whether the second skyrmion and its accompanied conical phase are originated from decoupling of coupled Cu skyrmion sublattices or it is related to new kind of spin skyrmion structure. A detailed reciprocal space map using the neutron scattering and scanning tunneling microscope studies are particularly required to shed light on this complex skyrmion behavior. Our findings open up a new pathway for further experimental and theoretical research to elucidate the exotic quantum topological skyrmion phases using chemical doping.

In summary, we have successfully synthesized and well characterized the high quality polycrystalline (Cu_1−x_Zn_x_)_2_OSeO_3_ (0 ≤ x ≤ 0.2) samples. Zn doped skyrmion exhibits the complex and rich phase diagram. The significant findings are: (1) The dopant Zn is favored to occupy the Cu(II) square pyramid crystallographic site. (2) The M-*T* and χ′_ac_-*T* are changed dramatically with the increase of Zn doping concentration. (3) The skyrmion phase shown in H-*T* phase diagram of Cu_2_OSeO_3_ is split with Zn doping as demonstrated in detailed H and *T* dependent χ′_ac_ data. (4) Second conical boundary extracted from the dχ′_ac_/dH vs. H curves accompanied by the second skyrmion phase. All these results suggest a interesting novel scenario and this unexpected observed appearance of second skyrmion and its conical boundary might be related to the way that the Zn doping manipulates the DM vector of the distorted Cu(II)O_5_ square pyramid through the influence of delicate magnetic interactions. These results point to a new direction of tuning the skyrmion lattice in Cu_2_OSeO_3_ by assorted chemical and atomic modification.

## Methods

In this study, polycrystalline samples of (Cu_1−x_Zn_x_)_2_OSeO_3_ (0 ≤ x ≤ 0.2) were prepared by solid-state reaction method. Nominal mixtures of high purity CuO, ZnO, and SeO_2_ powders were pressed into pellets. The pellets were sealed in an evacuated quartz tube and heated to a temperature range of 520 °C to 600 °C for 72 h, then slowly cooled over several hours to room temperature. This process was repeated at least twice with intermediate grinding. X-ray diffraction patterns show good quality of samples, with only a minor impurity phases appearing when x ≥ 0.06. Homogeneity of Zn distribution was analyzed with energy dispersive X-ray analysis, which indicated a uniform distribution of Zn throughout the sample (the supplementary material). Temperature and field dependent DC magnetization and AC susceptibility measurements were performed by a SQUID magnetometer (MPMS-XL7, Quantum Design). EXAFS *K*-edge experiments were carried out in transmission mode for Cu and fluorescence mode for Zn respectively at the 17C beamline in the National Synchrotron Radiation Research Center (NSRRC), Hsinchu, Taiwan.

## Additional Information

**How to cite this article**: Wu, H. C. *et al.* Unexpected observation of splitting of skyrmion phase in Zn doped Cu_2_OSeO_3_. *Sci. Rep.* **5**, 13579; doi: 10.1038/srep13579 (2015).

## Supplementary Material

Supplementary Information
