# Supplementary material for: Unexpected observation of splitting of skyrmion phase in Zn doped Cu2OSeO3
Source: Sci Rep. 2015 Sep 9;5:13579. doi: 10.1038/srep13579 (PMC4563258; doi:10.1038/srep13579)
Supplement: Supplementary Information [file srep13579-s1.pdf]

Unexpected observation of splitting of skyrmion phase in Zn doped Cu_2OSeO_3

H. C. Wu[1], T. Y. Wei[1], K. D. Chandrasekhar[1], T. Y. Chen[1], H. Berger[2] and H. D. Yang[1*]

[1]*Department of Physics, National SunYat-SenUniversity, Kaohsiung, 804 Taiwan*

[2]*Institute of Physics of Complex Matter, Ecole Polytechnique Federal de Lausanne, CH-1015 Lausanne, Switzerland*

* yang@mail.nsysu.edu.tw

Supplementary material:

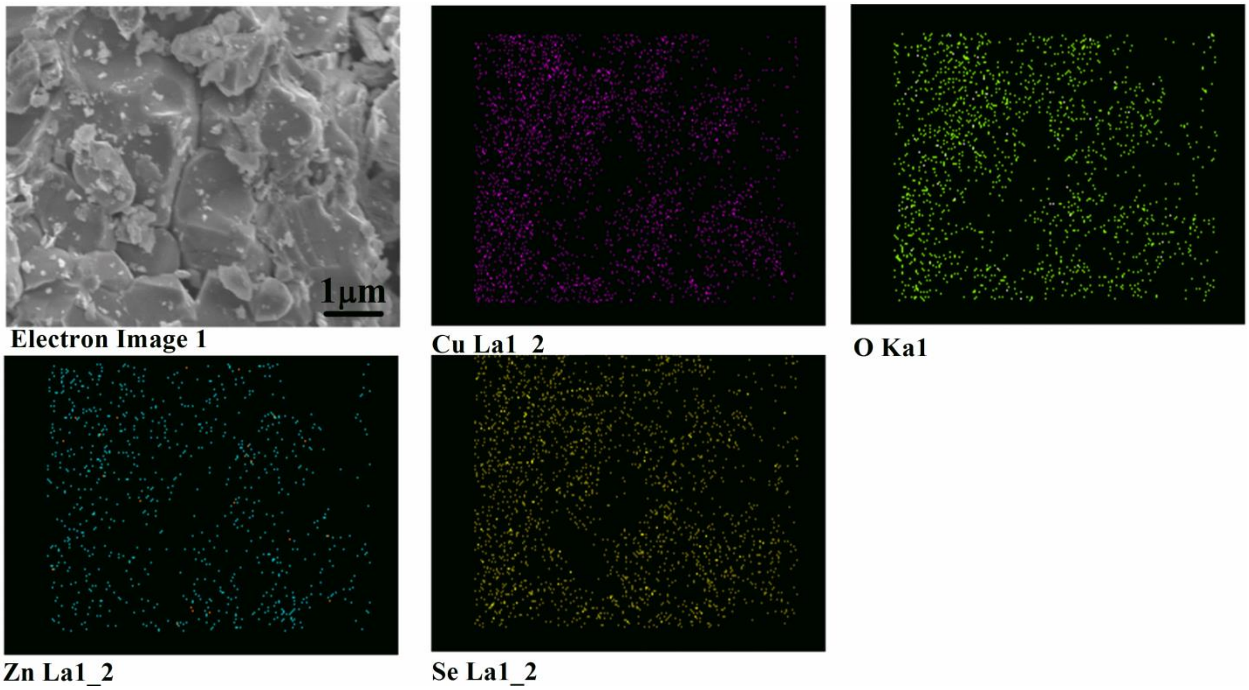

Figure 1: (color online). The elemental mapping by the energy dispersive X-ray analysis for $(Cu_{1-x}Zn_x)_2OSeO_3$ (x = 0.1) sample.

To verify the Zn homogeneity, we have performed the energy dispersive X-ray analysis (EDXA). Fig. 1 shows the elemental mappings of Zn, Cu, Se and O for the $(Cu_{0.9}Zn_{0.1})_2OSeO_3$ sample. As illustrate in the figures all the elements are uniformly distributed within the selected area of the sample, which indicates good homogenity of the sample. Moreover, elemental analysis on several parts of the sample (not shown here) is quantitatively similar, which ruled out the segregation or spinodal decomposition of Zn element.

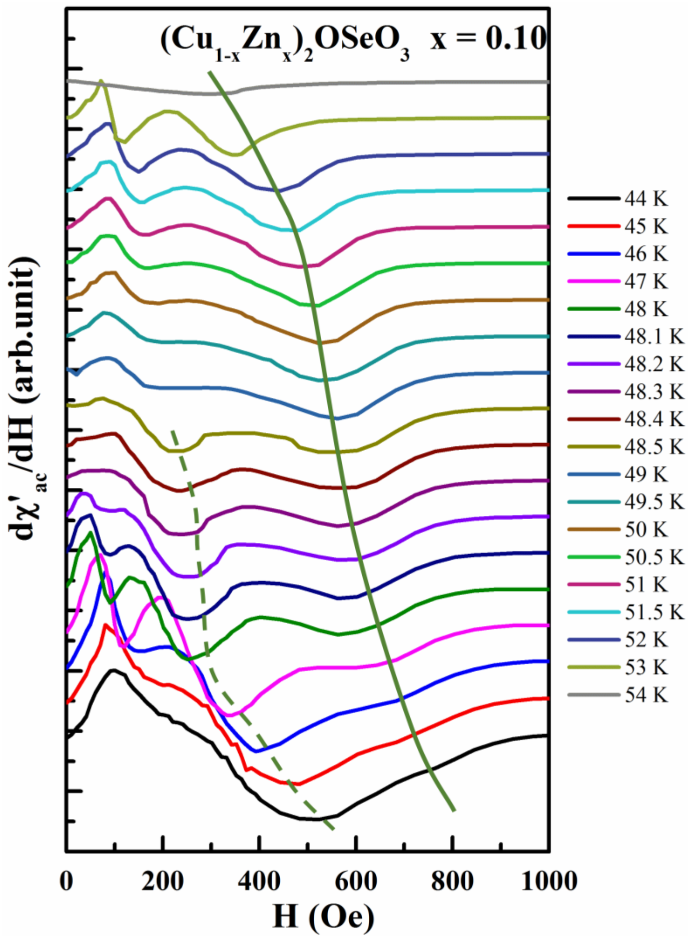

Figure: 2 (color online) $d\chi'_{ac}/dH$ vs. H curves for $(Cu_{1-x}Zn_x)_2OSeO_3$ (x = 0.1) at different temperatures. The solid and dashed green lines show the two conical phase boundaries respectively.

Fig. 2 displays the first derivative of χ'_{ac} with respect to H. As illustrated in figure, near to ferrimagnetic transition (54 K), a single inflection point has been observed (~ 320 Oe) in the $d\chi'_{ac}/dH$ vs. H curve. The dip is getting more stronger for low *T* curves, and this has been indicated as a conical phase boundary. Interestingly, for 44 K < *T* < 48.5 K, second inflection point appeared in the intermediate field range, and this dip has been denoted as the second conical phase boundary. The oscillatory signature at the low fields (H < 300 Oe) in both low 51

K ≤ T ≤ 53 K and high-temperatures 46 K ≤ T ≤ 48 K originated from the both the skyrmion lattices respectively.

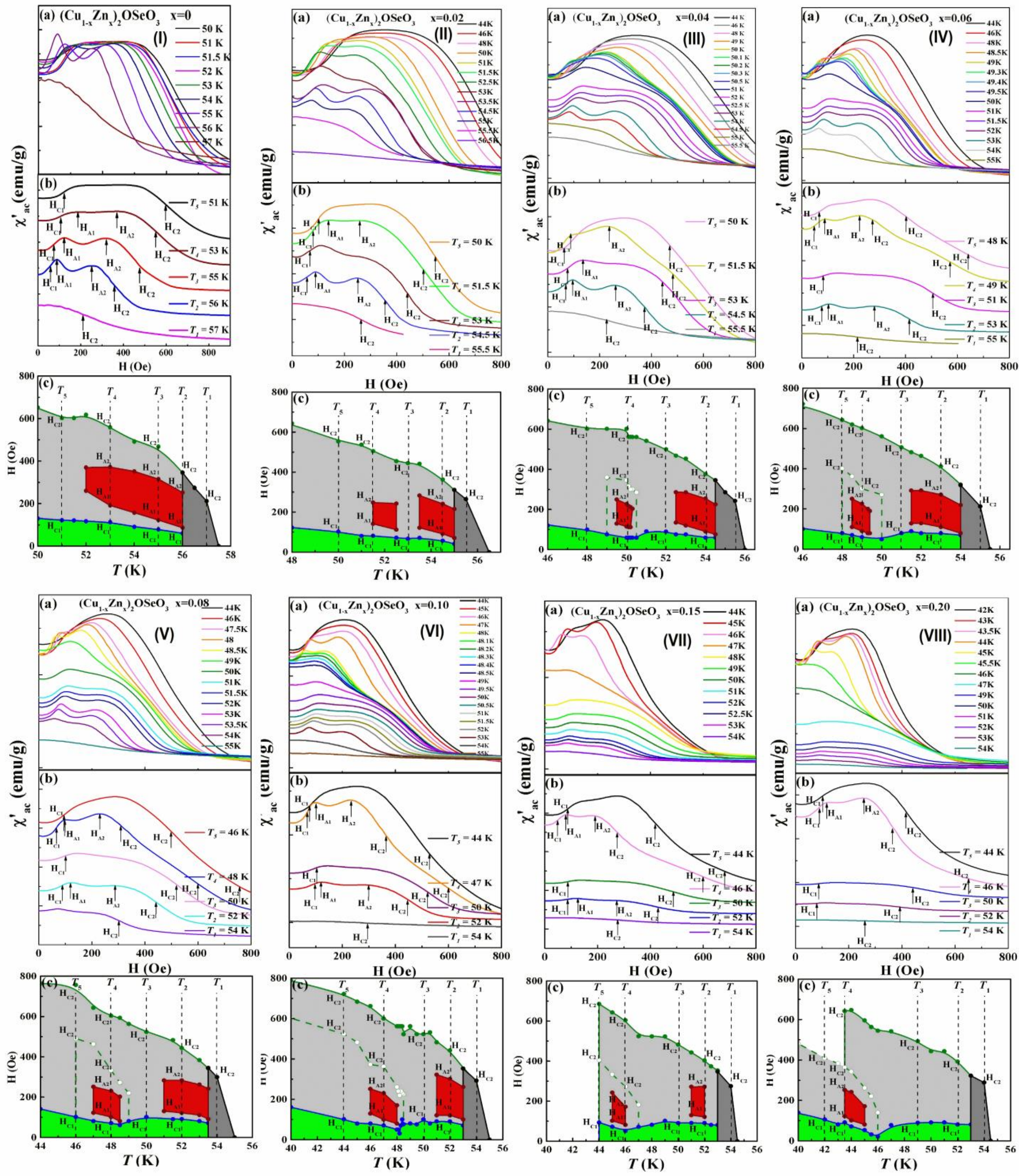

Figure 3: (color online). (a) χ'_{ac} vs. H plot for different temperatures. (b) Selected temperature curves from (a), but offset each other for easy comparison. The notations of H_{A1} and H_{A2} indicate skyrmion phase boundaries while H_{C1} and H_{C2} indicate conical phase boundaries respectively. The values of H_{A1} and H_{A2} are determined by the peaks while H_{C1} and H_{C2} are the inflection points in the first derivative of χ'_{ac} vs. H curves. (c) H vs. *T* phase diagram, for (I) x =0 (II) x =0.02 (III) x =0.04 (IV) x =0.06 (V) x =0.08 (VI) x =0.10 (VII) x =0.15 and (VIII) x =0.20 samples respectively.

Fig. 3 represents the χ'_{ac} vs. H and H-*T* phase diagram curves for different Zn doping concentration. For high Zn doping, the χ'_{ac} vs. H curves exhibit more complex behavior. That is a reappearance of skyrmion (oscillatory behavior) signature at low fields followed by the inflection points in $d\chi'_{ac}/dH$ vs. H curves at high magnetic fields. A more clear signature of this feature demonstrated by representive χ'_{ac} vs. H curves for selected temperatures as shown in Fig. 3 (b)'s of all the samples. The respective H-*T* phase diagram is shown in Fig. 3 (c)'s where the reappearance of second skyrmion is noticed which is denoted as a second red zone in the H-*T* phase diagrams.

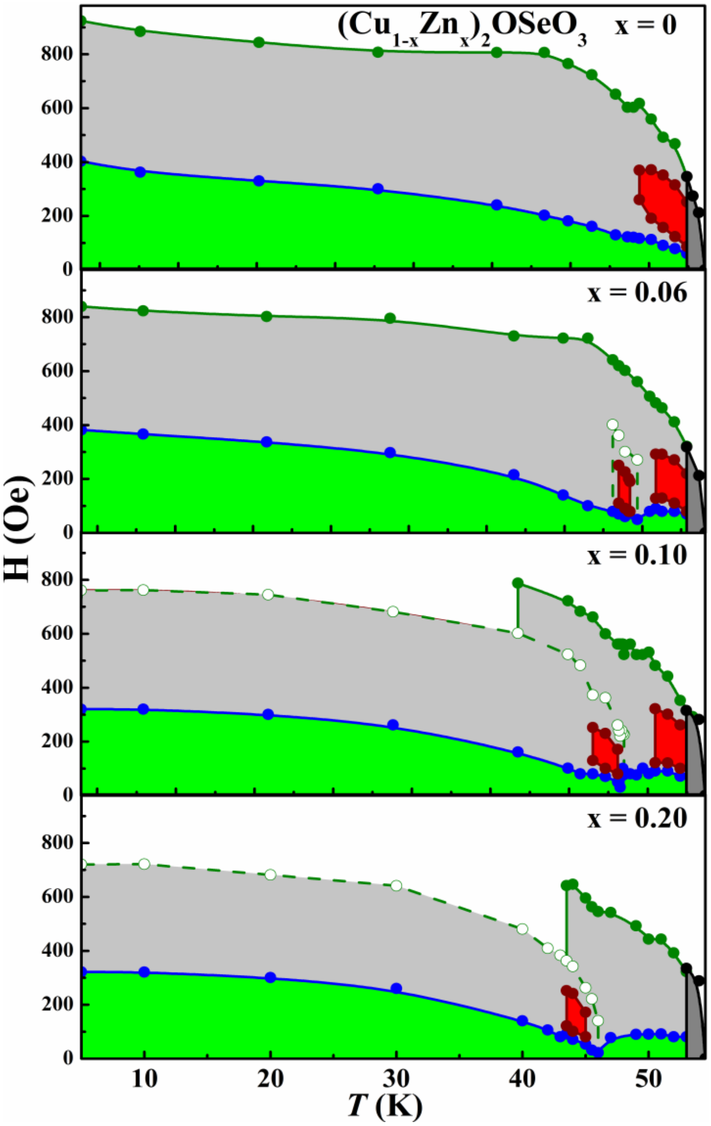

Figure 4: (color online). H-T phase diagrams of selected samples. Skyrmion zone indicated by red areas and solid and dashed green lines denote the two conical phase boundaries respectively.

Even though, our prime focus of the current manuscript is to understand the effect of Zn doping on the skyrmion phase. We have performed the χ'_{ac} vs. H measurements for selected samples up to 5 K to understand the low temperature H-T phase diagram. Fig. 4 shows the H-T phase diagram for selected samples. The H-T phase diagram does not exhibit any additional phases up

to 5 K. The conical and helical phase boundaries enhance with a decrease of *T*. The H-*T* phase diagram of parent Cu_2OSeO_3 itself exhibits very complex phases. Further, the influence of Zn doping on the several zones of H-*T* phase quite complex to understand from the present results. In fact, we are currently using small angle neutron scattering to look for the deeper insight of different zone of H-*T* phases upon Zn substitution.
